# 862 Use of a Dual Layer Biosynthetic Wound Matrix for Management of SJS/TEN

**DOI:** 10.1093/jbcr/iraf019.393

**Published:** 2025-04-01

**Authors:** Alexandra Lacey, Marah Kays, Charlotte Rogers

**Affiliations:** Regions Hospital Burn Center; University of Minnesota; Regions Hospital Burn Center

## Abstract

**Introduction:**

Steven-Johnson syndrome (SJS) and toxic epidermal necrolysis (TEN) are diseases in which there is an immune mediated necrosis of the epidermis causing life threatening sequelae. One of the challenges in treatment of SJS/TEN is management of massive fluid losses from this lack of epidermis. There is currently no consensus regarding the ideal wound care for these patients. Recently, a new dual layer biosynthetic wound matrix was made commercially available; this produce has the ability to act as a barrier on the wounds to help manage fluids, decrease pain with dressings, prevent infection, and promote wound healing.

**Methods:**

We present two case reports of the use of a dual layer biosynthetic wound matrix in the setting of biopsy proven SJS/TEN at an ABA verified burn center in the year 2024.

**Results:**

Patient A is a 23 year old woman with biopsy proven TEN with sloughing of 30% TBSA plus ocular and mucosal involvement (including gastrointestinal). She underwent placement of the biosynthetic wound matrix on post injury day 9. Pre-operatively she had been requiring over 250 mL/hr of lactated ringers. By post-operative day 2 she was undergoing active diuresis and no longer needing volume support. She discharged on post operative day 16 with over 90% of her wounds completely closed.

Patient B is a 5 year old girl with biopsy proven TEN with 86% TBSA sloughing. She underwent placement of the biosynthetic wound matrix on hospital day 5. Pre operatively she was routinely soaking her dressings with wound drainage and requiring fluid resuscitation. By post operative day two she no longer required intravenous fluids. Additionally, the amount of pain meds required for dressing changes dramatically decreased. She discharged on post operative day 43 with over 80% of her wounds healed.

**Conclusions:**

The use of the novel biosynthetic wound matrix in both patients with TEN resulted in abrupt decrease in the requirement for ongoing fluid resuscitation. One patient had an infection develop under the matrix that was treated with debridement and antibiotics. Both patients had excellent evidence of wound healing and were able to discharge directly to home after this significant critical illness.

**Applicability of Research to Practice:**

This paper outlines the first published use of a novel biosynthetic wound matrix in patients with SJS/TEN.

**Funding for the Study:**

No external funding was used for this project.

